# Decreased platelet responsiveness to clopidogrel correlates with *CYP2C19* and *PON1* polymorphisms in atherosclerotic patients

**DOI:** 10.1590/1414-431X20165660

**Published:** 2017-01-09

**Authors:** J.F.M. Marchini, M.R. Pinto, G.C. Novaes, A.V. Badran, R.B. Pavão, G.L. Figueiredo, I.M. Lago, M.O. Lima-Filho, D.C. Lemos, M. Tonani, C.M. Antloga, L. Oliveira, J.C. Lorenzi, J.A. Marin-Neto

**Affiliations:** 1 Unidade de Hemodinâmica e Cardiologia Intervencionista, Divisão de Cardiologia, Departamento de Clínica Médica, Faculdade de Medicina de Ribeirão Preto, Universidade de São Paulo, Ribeirão Preto, SP, Brasil; 2 Departamento de Odontologia, Universidade de Uberaba, Uberaba, MG, Brasil; 3 Divisão de Hematologia, Departamento de Clínica Médica, Faculdade de Medicina de Ribeirão Preto, Universidade de São Paulo, Ribeirão Preto, SP, Brasil; 4 Departamento de Genética, Faculdade de Medicina de Ribeirão Preto, Universidade de São Paulo, Ribeirão Preto, SP, Brasil

**Keywords:** Platelet function tests, Single nucleotide polymorphism, Percutaneous coronary intervention, Aspirin, Clopidogrel

## Abstract

Clopidogrel and aspirin are the most commonly used medications worldwide for dual antiplatelet therapy after percutaneous coronary intervention. However, clopidogrel hyporesponsiveness related to gene polymorphisms is a concern. Populations with higher degrees of genetic admixture may have increased prevalence of clopidogrel hyporesponsiveness. To assess this, we genotyped *CYP2C19*, *ABCB1*, and *PON1* in 187 patients who underwent percutaneous coronary intervention. Race was self-defined by patients. We also performed light transmission aggregometry with adenosine diphosphate (ADP) and arachidonic acid during dual antiplatelet therapy. We found a significant difference for presence of the CYP2C19*2 polymorphism between white and non-white patients. Although 7% of patients had platelet resistance to clopidogrel, this did not correlate with any of the tested genetic polymorphisms. We did not find platelet resistance to aspirin in this cohort. Multivariate analysis showed that patients with *PON1* and *CYP2C19* polymorphisms had higher light transmission after ADP aggregometry than patients with native alleles. There was no preponderance of any race in patients with higher light transmission aggregometry. In brief, *PON1* and *CYP2C19* polymorphisms were associated with lower clopidogrel responsiveness in this sample. Despite differences in *CYP2C19* polymorphisms across white and non-white patients, genetic admixture by itself was not able to identify clopidogrel hyporesponsiveness.

## Introduction

Dual antiplatelet therapy (DAPT) reduces thrombosis and major ischemic cardiovascular events (1,2) in patients with coronary artery disease treated with percutaneous coronary intervention (PCI) (3,4). DAPT consists of a combination of acetylsalicylic acid (ASA) and clopidogrel (or one of the newer drugs acting at the P2Y_12_ platelet receptor). Although inhibition of platelet activation is less variable among patients with newer P2Y_12_ receptor blockers, clopidogrel is still important because of its availability as a generic drug and lower cost. Furthermore, in Brazil it is provided free of charge by the national Unified Health System.

Despite routine use of DAPT, there are still cases of stent thrombosis and acute vessel closure after PCI, which may be attributed to resistance to one or both components of the DAPT regimen (5,6). In addition, resistance to antiplatelet drugs is associated with worse outcomes (7,8). There are multiple factors associated with antiplatelet drug resistance, including diabetes mellitus, congestive heart failure and obesity, but also gene polymorphisms, which have been linked to clopidogrel hyporesponsiveness and stent thrombosis (8–10). Known genes that affect clopidogrel response include *ABCB1*, *CYP2C19*, and *PON1*. The product of *ABCB1* is an efflux transporter p-glycoprotein expressed in the gut that modulates clopidogrel absorption. CYP complex proteins activate clopidogrel through a two-step metabolic process and CYP2C19 participates in both steps (11). *PON1* encodes the enzyme paraoxonase-1, which participates in the esterification of clopidogrel and its subsequent inactivation (11).

Although previous studies have described genotype frequencies for *CYP2C19* and *ABCB1* in populations with a high degree of genetic admixture, these were conducted in healthy subjects (12–14). Therefore, we examined all three genes that affect clopidogrel response and analyzed the effects of clopidogrel in a prospective cohort of high-risk patients treated with PCI. We also examined platelet responsiveness to ASA and clopidogrel.

## Material and Methods

### Study design and participants

The design and rationale of the SPARC (Sequence Variation in Platelet Aggregation in Response to Clopidogrel and acetylsalicylic acid) study have been described elsewhere (15). Briefly, this is a single-center, observational, prospective trial that enrolled patients from December 2009 to January 2011 who underwent PCI and had no contraindications to ASA or clopidogrel. We targeted an enrollment of 200 patients that would allow detection of a rare allele (frequency <5%) with a 95% confidence interval. This study was approved by the Ribeirão Preto Medical School institutional ethics committee (SISNEP-CAAE 0153.0.004.000-09), and was registered in the Brazilian Clinical Trials Registry as RBR-6n87rs. All patients agreed to participate and provided written informed consent.

At least 6 h prior to PCI, patients received a loading dose of 300 mg ASA and 300 mg clopidogrel. After the procedure, we prescribed clopidogrel therapy (75 mg/day) for 30 days when bare metal stents had been used, and for 1 year when drug-eluting stents had been implanted. We also recommended continuous use of ASA (100 mg/day) after PCI. We recorded baseline data and clinical follow-up at 1, 3, 6, and 12 months.

Blood samples were collected from all patients on enrollment for genotyping. We also performed light transmission aggregometry (LTA) after PCI. After the recommended period of DAPT, we measured control LTA on two occasions, first with patients having discontinued clopidogrel use for at least 1 week and second with patients 1 week off ASA and on clopidogrel (at least 6 months after PCI). We defined major adverse cardiac events (MACE) as a composite of target vessel revascularization, other revascularization, myocardial infarction (MI), and death. We adopted the first universal definition of MI (16) and the ARC definition for stent thrombosis (17). The study was not powered to detect differences in MACE across genotypes.

### Genotyping by TaqMan polymerase chain reaction (PCR)

Using the FlexiGene DNA Kit (QIAGEN, Germany) in accordance with manufacturer instructions, we extracted genomic DNA from peripheral blood (428 µL) collected in ACD tubes (BD, USA). We determined genotype using the Drug Metabolism Genotyping Assays (IDs C_25986767_70, C_27861809_10, C_7586657_20, and C_2548962_20; Life Technologies, USA) for CYP2C19*2 (rs4244285), CYP2C19*3 (rs4986893), *ABCB1* C3435T (rs1045642), and *PON1* Q192R (rs2158155), respectively.

### Light transmission aggregometry

We collected peripheral blood for LTA in 3.2% sodium citrate tubes and centrifuged samples at 1000 *g* for 10 min at room temperature to separate platelet-rich plasma from platelet-poor plasma. We diluted the platelet-rich plasma to 200,000 platelets/µL and performed aggregometry in a Helena AggRAM 1484 system, with 5 µM adenosine diphosphate (ADP) to test clopidogrel responsiveness and 1 mM arachidonic acid to test ASA responsiveness. In both assays, measurements were obtained 5 minutes after agonist addition. We defined cutoff levels for high on-treatment platelet reactivity (HTPR) as 86% for clopidogrel and 80% for ASA based on previous trials showing increased adverse events above these thresholds (8,18).

### Statistical analysis

Data are reported as percentages for categorical variables and as means±SD for continuous variables.

We used the chi-square test for comparative analysis of allelic and genotypic frequencies of the gene polymorphisms and of predicted frequency according to ethnicity. The Wilcoxon rank-sum test was used to evaluate association of increased LTA with each polymorphism, as well as to test for trend across ranked groups (19). We tested the association of LTA with clinical variables and the presence of specific polymorphisms using simple and multiple linear regression. The level of significance for hypothesis testing was set at 0.05.

The Stata 12 software package (StataCorp, USA) was used for all statistical analyses.

## Results

We evaluated 197 patients for potential participation in this study and enrolled 190. Of these, 7 patients did not undergo PCI and three others withdrew consent. Table 1 presents baseline data for the 187 remaining patients (15). Most patients (82%) were self-identified as white, 15% were self-identified as brown, and 3% as black.

**Table:**
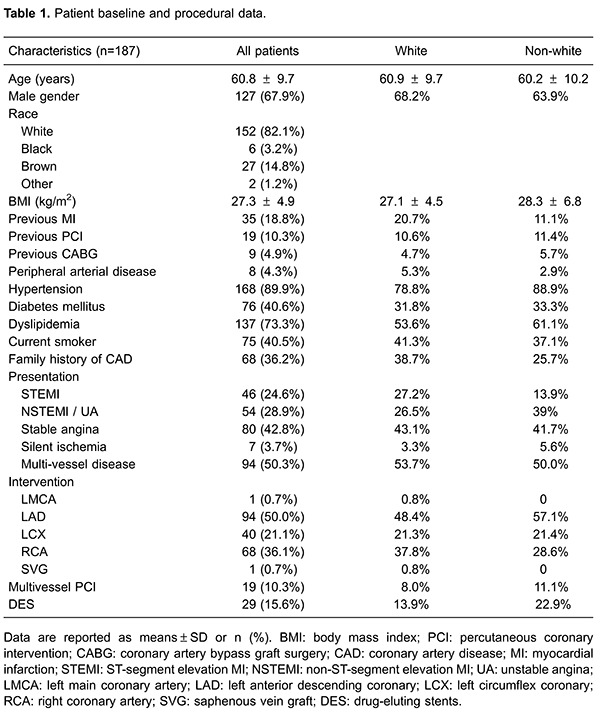

We assessed four single nucleotide polymorphisms (SNP) in three genes involved in the metabolism and activation of clopidogrel: *ABCB1*, *PON1*, and *CYP2C19*. The frequency of each genotype is reported in Table 2. CYP2C19*3 was not present in any of the patients enrolled in this study. There was a significant difference of CYP2C19*2 frequency by race (P=0.039). Non-white patients were less likely to be heterozygous and three times more likely to carry a homozygous mutant. Furthermore, these patients were not in Hardy-Weinberg equilibrium (P<0.05), which could be explained by population migration. The other SNPs had no significant difference across races.

**Table:**
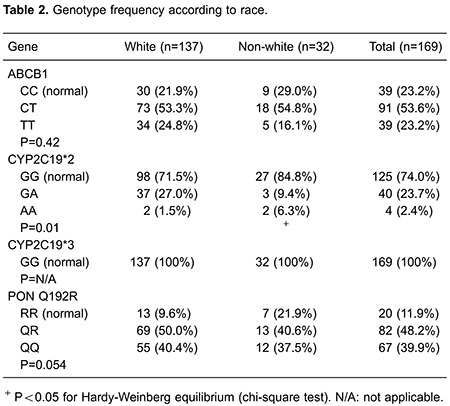

To examine the effect of clopidogrel and ASA treatment, LTA was performed in 53 patients (28.3% of all patients). Mean LTA with ADP stimulation changed from 84.9±8.5 at the control time-point (off clopidogrel) to 66.6±15.1 when on DAPT, while mean LTA with arachidonic acid stimulation changed from 51.2±36.7 at the control time-point (off ASA) to 13.7±15.0 when patients were on DAPT. Overall, 6.9% of patients had HTPR to clopidogrel, while none had HTPR to ASA. We did not find an association of HTPR with any of the tested SNPs, alone or in combination.

When compared to normal homozygous patients, patients carrying a copy of a CYP2C19*2 allele had increased LTA(ADP) despite clopidogrel use (71.2±14.5 *vs* 64.4±14.4, P=0.044). Carriers of at least one *PON1* Q allele also had increased LTA(ADP) despite clopidogrel therapy (68.2±14.5 *vs* 58.8±13.9, P=0.047).

We performed a pooled analysis of *CYP2C19* and *PON1* alleles. We divided patients into three groups: those with no mutation in either gene (8.5% of patients); those with a mutation in one of the genes (70.5%); and those with a mutation in both genes (21%). Patients who had only normal copies of both genes had a mean LTA(ADP) of 50.5±12.9. The largest group, comprising patients who had only one of the genes bearing a polymorphism, had a mean LTA(ADP) of 66.9±14. Finally, in patients who had both genes altered, the mean LTA(ADP) was 71.9±16.7. We found a trend between increased LTA and more altered alleles (P=0.006 for the trend). Other gene combinations had no detectable trend for association with LTA.

**Figure 1:**
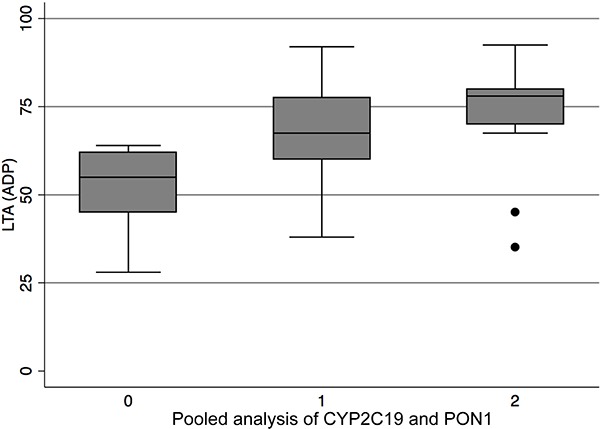
Box-whisker plot of percentage of light transmission aggregometry (LTA) with adenosine diphosphate (ADP) stimulation on dual antiplatelet therapy with clopidogrel and acetylsalicylic acid according to pooled analysis of *CYP2C19* and *PON1*. For this analysis, patients were stratified into three groups: those with no mutation in either gene were assigned a value of (0); those with a mutation in only one gene (either in one or two copies) were assigned a value of (1); and those with mutations in both genes were assigned a value of (2).

Multiple clinical factors are linked to the LTA response to clopidogrel, as reported elsewhere (20–22). Table 3 lists the results of univariate linear regression analysis for LTA(ADP) in our sample. Race was not a predictor of LTA. Only dyslipidemia (P=0.02) and pooled analysis of mutant alleles in the *CYP2C19* and *PON1* loci (P=0.005) correlated with variations in LTA. On multivariate analysis, only the loci combination remained significant. Figure 1 shows LTA values according to SNPs in *CYP2C19* and *PON1*.

**Table:**
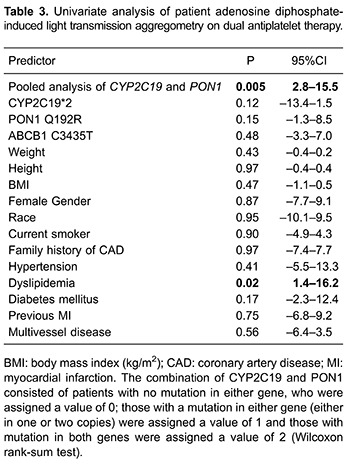

We followed patients for a mean of 377±137 days. After the index procedure, 7 patients had an additional planned PCI. There were 19 (10.2%) MACEs, including 4 cardiac deaths, 6 non-fatal MIs, 1 stroke, 4 target lesion revascularizations, and 3 coronary interventions in non-target vessels, as shown in Table 4. There was one episode of MI due to definite stent thrombosis, in a patient in whom neither genotyping nor LTA had been performed. There were no associations of nucleotide polymorphisms, measured LTAs, baseline clinical characteristics, or risk factor reductions with MACE.

**Table:**
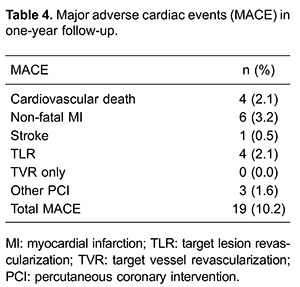

## Discussion

In this study, we genotyped four polymorphisms in genes related to clopidogrel responsiveness in a prospective cohort of 187 patients with coronary artery disease who had undergone PCI. *ABCB1* genotype frequencies in our sample were significantly different (P=0.014) from those reported previously in healthy Brazilian volunteers (13). This may reflect heterogeneous genetic admixtures of different populations, since our study enrolled patients from the Southeast region of Brazil, while the other study had a much broader sample recruited from many regions of the country. CYP2C19*2 genotypes were similar to those described in previous reports, even respecting racial differences (13). The genotype frequencies of *PON1* among patients who were self-identified as white in our study were similar to those of the HapMap-CEU population, which includes Americans of Northern and Western Europe ancestry, but frequencies in our non-white subsample did not match. Although numerically different, *PON1* genotype frequencies in our study were not statistically different between white and non-white patients.

We tested for correlation between genotypes known to influence response to clopidogrel and results of platelet function testing, as assessed by LTA with the agonists ADP and arachidonic acid. We did not find associations of any of the tested SNPs with HTPR for clopidogrel, but both the CYP2C19*2 allele and the *PON1* Q allele were associated with increased LTA (ADP), with a significant trend (P=0.006) in patients who had both altered alleles. This was also the sole significant predictor on multivariate analysis.

Considering that non-white patients have an increased prevalence of the CYP2C19*2 gene, we would expect a higher LTA in non-white patients, which we did not find. Non-white patients had numerically higher rates of the normal PON1 R allele, which may have balanced out the effects of the CYP2C19*2 gene. Suspecting increased resistance to clopidogrel solely on the basis of race may not be adequate for therapeutic decision-making.

The very hypothesis of *PON1* modulation of platelet function is controversial. Our work is in line with the initial publication showing this relationship (10), which has not been reproduced in other reports (23–25).

In conclusion, just over 20% of patients in our population had alterations in at least two genes implicated in clopidogrel activation. Although non-white patients had an increased prevalence of CYP2C19*2, race did not predict clopidogrel hyporesponsiveness. It is reassuring that the patients who had HTPR to clopidogrel did not have HTPR to ASA.

Although other studies have demonstrated that specific genotypes are associated with worse outcomes, genotyping alone is not enough to predict clopidogrel hyporesponsiveness. On the one hand, determining which genes are altered in an individual patient can help elucidate mechanisms of resistance, but it is unlikely to have an impact on care. Platelet function tests, on the other hand, identify clopidogrel hyporesponsiveness not only from SNPs but also from clinical factors, such as acute coronary syndrome (26). This mechanism may explain why prasugrel (27) and ticagrelor (28) have been shown to be associated with fewer MACEs than clopidogrel in the setting of acute coronary syndrome. Nevertheless, to date, no study has shown that monitoring of platelet function to adjust antiplatelet management improves outcomes (29,30).

### Limitations

We did not measure other genotypes that may be important to the interpretation of platelet resistance to clopidogrel, such as CYP2C19*17. Furthermore, our analysis of LTA was limited to 28.3% of all patients and was performed with 5 µM of ADP. Although recent recommendations advise a lower concentration of this agonist, our protocol was considered acceptable at the time of the study (31–33).
